# Sucrose synthase gene family in *Brassica juncea*: genomic organization, evolutionary comparisons, and expression regulation

**DOI:** 10.7717/peerj.10878

**Published:** 2021-03-09

**Authors:** Mengyao Li, Qi He, Ying Huang, Ya Luo, Yong Zhang, Qing Chen, Yan Wang, Yuanxiu Lin, Yunting Zhang, Zejing Liu, Xiao-Rong Wang, Haoru Tang

**Affiliations:** 1 College of Horticulture, Sichuan Agricultural University, Chengdu, China; 2 College of Agriculture and Forestry Science, Linyi University, Linyi, China; 3 Institute of Pomology and Olericulture, Sichuan Agricultural University, Chengdu, China

**Keywords:** Sucrose synthase, *Brassica juncea*, Phylogenetic analysis, Syntenic analysis, Gene expression

## Abstract

Sucrose synthase (SUS) plays an important role in sucrose metabolism and plant development. The SUS gene family has been identified in many plants, however, there is no definitive study of SUS gene in *Brassica juncea*. In this study, 14 SUS family genes were identified and comprehensively analyzed using bioinformatics tools. The analyzed parameters included their family member characteristics, chromosomal locations, gene structures and phylogenetic as well as transcript expression profiles. Phylogenetic analysis revealed that the 14 members could be allocated into three groups: SUS I, SUS II and SUS III. Comparisons of the exon/intron structure of the mustard SUS gene indicated that its structure is highly conserved. The conserved structure is attributed to purification selection during evolution. Expansion of the SUS gene family is associated with fragment and tandem duplications of the mustard SUS gene family. Collinearity analysis among species revealed that the SUS gene family could be lost or mutated to varying degrees after the genome was doubled, or when *Brassica rapa* and *Brassica nigra* hybridized to form *Brassica juncea*. The expression patterns of *BjuSUSs* vary among different stages of mustard stem swelling. Transcriptomics revealed that the *BjuSUS01-04* expression levels were the most elevated. It has been hypothesized that they play an important role in sucrose metabolism during stem development. The expression levels of some *BjuSUSs* were significantly up-regulated when they were treated with plant hormones. However, when subjected to abiotic stress factors, their expression levels were suppressed. This study establishes SUS gene functions during mustard stem development and stress.

## Introduction

Sucrose synthase (Sucrose Synthase, EC2. 4. 1. 13, SUS) is a tetramer composed of subunits with a molecular weight of approximately 83 kD to 100 kD. It is involved in sucrose metabolism (Tanase & Yamaki, 2000; Chalivendra et al., 2007). It catalyzes sucrose synthesis and decomposition. Although sucrose synthase is a reversible enzyme, it converts sucrose and UDP into UDP-glucose and fructose (Nguyen-Quoc & Foyer, 2001). During different stages of plant growth and development, sucrose, fructose and glucose levels are constantly altered. High glucose levels during the early stages are a source of carbon that is required during the rapid growth of plant tissues. This allows plant cells to maintain a high osmotic pressure to ensure adequate water absorption. The gradual increase in sucrose levels after water absorption enhances sugar accumulation in the plant tissues (Dai et al., 2011). Sucrose synthase is involved in sucrose synthesis and decomposition. Therefore, the biological activities and gene expression profiles of SUS vary among different stages of plant growth and development. Studies have reported that SUS is also important in the synthesis of starch (Heim et al., 1993) and fiber cell wall (Ruan, Llewellyn & Furbank, 2003), fruit development (Wang, Smith & Brenner, 1994) as well as in plant stress (Jha & Dubey, 2004).

Since the identification of the first SUS gene in wheat germs, it has been cloned in various crops such as potato, carrot, corn, citrus and *Arabidopsis*. The SUS gene family has been identified and reported in several plants. Five SUS genes have been identified in six in *Arabidopsis*, six in rice, three in maize, 15 in *Populus* and 14 in tobacco (An et al., 2014; Baud, Vaultier & Rochat, 2004; Duncan, Hardin & Huber, 2006; Hirose, Scofield & Terao, 2008; Wang et al., 2015). The SUS gene family is involved in the regulation of multiple pathways throughout the growth cycle of the plant. It has been revealed that when the *SUS4* gene is highly expressed during starch synthesis in potato tubers and the activity of SUS enzyme is significantly elevated, there is an increase in ADPG and UDPG levels and a nearly double starch yield (Baroja-Fernandez et al., 2009). Several SUS genes (*GhSUS1A-GhSUS8*) are involved in cellulose accumulation and secondary wall thickening of the tetraploid cotton xylem. They are important during fiber elongation at different stages of cotton growth (Ruan, Llewellyn & Furbank, 2003).

The tuber mustard is a stem variety of the mustard in which enlarged fleshy stems are widely consumed while fresh and used for mustard processing. During the growth and development of the tumorous stem, the base of the petiole is metamorphosed to form 1–5 tubercular bulges that laterally expand with stem swelling. The genetic analysis of the shape of the swollen stem showed that its shape was mainly controlled by two major genes and polygenes, and there were obvious additive, dominant and epistatic genetic effects (Liu et al., 2014). The tumor-like swelling of the tuber mustard is closely correlated with its yield and quality. Sugar production and transportation in the mustard accumulate in the stem, enhancing its expansion. Sucrose synthase gene functions during stem swelling have not been established. It is, therefore, necessary to determine the structure, expression patterns and evolutionary relationship of SUS gene family in tuber mustard.

Based on the published sequencing results of the mustard genome, we identified 14 SUS genes in the allotetraploid mustard. Bioinformatic tools were then used to identify and characterize the mustard SUS gene in the whole genome of the mustard. In addition, the expression patterns of each member of the mustard SUS gene family in the process of stem swelling and their potential roles in response to various hormones and abiotic stresses were determined. These results provide a basis for further research on the potential functions of each mustard SUS gene, especially during the development of mustard stems.

## Materials and Methods

### Characterization and phylogenetic analysis of SUS genes in the *B. juncea* genome

The *B. juncea* genome sequence data (V 1.5) used in our study are available within the *Brassica* database (http://brassicadb.org/brad/). Using BLAST (https://blast.ncbi.nlm.nih.gov/Blast.cgi) with the expectation value of *E* > 1*e*^−10^, the amino acid sequences of SUS genes in *Arabidopsis* were used as query sequences to identify homologous genes. All the non redundant gene sequences encoding complete amino acid sequences were considered as *BjuSUS* genes. Sequence information of 14 *BjuSUS*s were submitted to NCBI website, with accession numbers from MW370524–MW370537.

Amino acid numbers, their molecular weights, predicted theoretical isoelectric points (pI), exon numbers, and subcellular localization were predicted using the ProtParam tool (http://www.expasy.org/tools/protparam.html). Amino acid sequences were aligned using ClustalX (2.1), while MEGA V6.06 was used to construct a phylogenetic tree based on the neighbor joining (N–J) method. A bootstrap value of 1,000 was used to test the reliability of the obtained trees (Kumar et al., 2008). The specific conserved domains in the SUS family were searched on InterProScan (http://www.ebi.ac.uk/Tools/InterProScan/), and the conserved domains were predicted by Pfam (http://pfam.xfam.org/search/sequence) (Sonnhammer, Eddy & Durbin, 1997). The GO (Gene Ontology, http://geneontology.org/) annotations of the BjuSUS proteins were analyzed using the Blast2GO (https://www.blast2go.com/). TBtools (https://github.com/CJ-Chen/TBtools) were used to generate the genetic structures of the SUS family while MEME (http://meme-suite.org/tools/meme) was used to identify conserved motifs (Bailey et al., 2009). The program Clustal W2 was used to calculate the similarity/identity of amino acid sequences. Subcellular localization analysis was performed using WOLF PSORT (https://wolfpsort.hgc.jp/) and TargetP (http://www.cbs.dtu.dk/services/TargetP/). Gene pair collinearity was determined using the MCScanX software (http://chibba.pgml.uga.edu/mcscan2/) (Wang et al., 2012) and then plotted using the Circos software. All cis-regulatory elements in the gene promoter are available on the online PlantCARE website (http://bioinformatics.psb.ugent.be/webtools/plantcare/html/).

### Plant materials, phytohormone and abiotic treatments

The experimental samples used to analyze the expression patterns at the developmental stage (2, 4, 6 and 8 cm) were obtained from the four stages of swollen stems of the tuber mustard variety ‘Fuza No.2’. All samples were grown in pots in a controlled growth environment. For hormonal and abiotic stress treatment, 4 week old plants with 5–6 true leaves were used in the experiments. The seedlings were separately sprayed with auxin (IAA) (10 μM), abscisic acid (ABA) (75 μM), salicylic acid (SA) (0.5 mM) and gibberellin (GA) (50 μM) and stored for 24 h. Abiotic stress was simulated by low temperature (4 °C), drought treatment (10% PEG8000), NaCl treatment (400 mM) and high temperature treatment (38 °C). All treatments were set to three biological replicates. After 12 h, the samples were obtained and immediately frozen in liquid nitrogen. They were then stored at −80 °C.

### RNA extraction and cDNA synthesis

Total RNA was extracted from stems at different growth stages by using the RNA prep Pure Plant kit (Tiangen, Beijing, China). The extracted RNA was spectrophotometrically quantified and electrophoresed on a 1.0% agarose gel to verify its integrity. First-strand cDNA was synthesized from 1.0 µg of total RNA using the PrimeScriptTM RT Reagent Kit with gDNA Eraser (TaKaRa, Tokyo, Japan). All cDNA samples were diluted 1:10 with RNase-free water.

### Expression analysis and Quantitative real-time PCR verification

The data obtained from the transcriptome sequencing of the different developmental stages of the stem tumor mustard variety ‘Fuza No.2’ (2, 4, 6 and 8 cm in diameter, respectively) was submitted to NCBI under the accession number SRP151320. The FPKM value was used to determine the expression abundance of the SUS gene during different developmental stages of *B. juncea*. The gene expression heat map was plotted using TBtools.

Quantitative real-time PCR (qRT-PCR) was performed using the Bio-Rad CFX96TM real-time PCR System at the standard mode with the 2×T5 Fast qPCR Mix (SYBRGreenI) (TsingKe, Beijing, China). All experiments were performed in triplicate. To normalize gene expression, *UBC* was used as the internal control (Li et al., 2020). The relative expression levels of *BjuSUSs* were calculated using the 2^−ΔΔCT^ method (Pfaffl, 2001). Primers were designed using Primer Premier 5.0 software. The primer sequences used in this study are listed in Table S1.

### Functional divergence analysis

Diverge 3.0 was used to perform type I and type II functional disproportionation analysis by the phylogenetic tree of the SUS proteins. Both type I and type II functional divergence occurred after gene replication. Type I functional divergence leads to specific amino acid selective changes, that is, changes in evolutionary rates, and its coefficient θ_I_ fluctuates between 0 and 1. This fluctuation reflects the functional divergence between gene categories from weak to strong. Type II functional divergence indicates that there are site-specific shifts of amino acid properties between subgroups (Gu, 2006). A high Qk (posterior probability) value indicates that the evolution rate or site-level physicochemical amino acid properties between the two clades are highly likely to be different. If Qk > 0.9, it is inferred that the amino acid position may exhibit functional divergence after SUS gene replication (Xun & Kent, 2002).

### Analysis of non-synonymous mutation rate to synonymous mutation rate ratios

The homologous gene pair of the *BjuSUS* gene family was identified using the OrthoMCL software (https://orthomcl.org/orthomcl/). The non-synonymous mutation rate (Ka), synonymous mutation rate (Ks) and Ka/Ks ratio of each pair of homologous genes were calculated using the Ka/Ks Calculator software. If Ka/Ks > 1, the gene is said to have been subjected to positive selection pressure; Ka/Ks = 1 indicates neutral selection; Ka/Ks < 1 is considered to have purification selection.

## Results

### Identification and analysis of BjuSUSs

Fourteen genes encoding BjuSUS proteins were identified and renamed *BjuSUS01* through *BjuSUS14*. Detailed information on genomic positions, coding region lengths, exon numbers, subcellular localizations and the corresponding proteins are summarized in Table S2. The open reading frame (ORF) size of *BjuSUSs* ranged from 3,447 (*BjuSUS04*) to 9,068 bp (*BjuSUS10*). They coded for proteins with a length that ranged from 755 to 905. The molecular mass of the fourteen proteins ranged from 85.42 to 102.93 kD, whereas their theoretical PI was between 5.67 (BjuSUS01) and 6.36 (BjuSUS13). These findings show that the BjuSUS protein is a weakly acidic protein. Subcellular localization prediction revealed that, except for BjuSUS07 and BjuSUS08 that are located in the mitochondria, the remaining BjuSUSs are located in the cytoplasm.

Multiple sequence alignment revealed a high level of similarity between the amino acid (52.08–99.02%) sequences (Table S3). The highest percentage of amino acid sequence identity was found between BjuSUS09 and BjuSUS10 (99.02%). A high pairwise identity of amino acid sequences was found in BjuSUS01/02, BjuSUS01/04, BjuSUS07/08 and BjuSUS13/14, ranging from 97.01% to 98.64%. The most of the sequences showed less than 80% amino acid sequence identity. The lowest sequence identity was found between BjuSUS04 and BjuSUS09 with 52.08% amino acid sequences.

**Figure 1:**
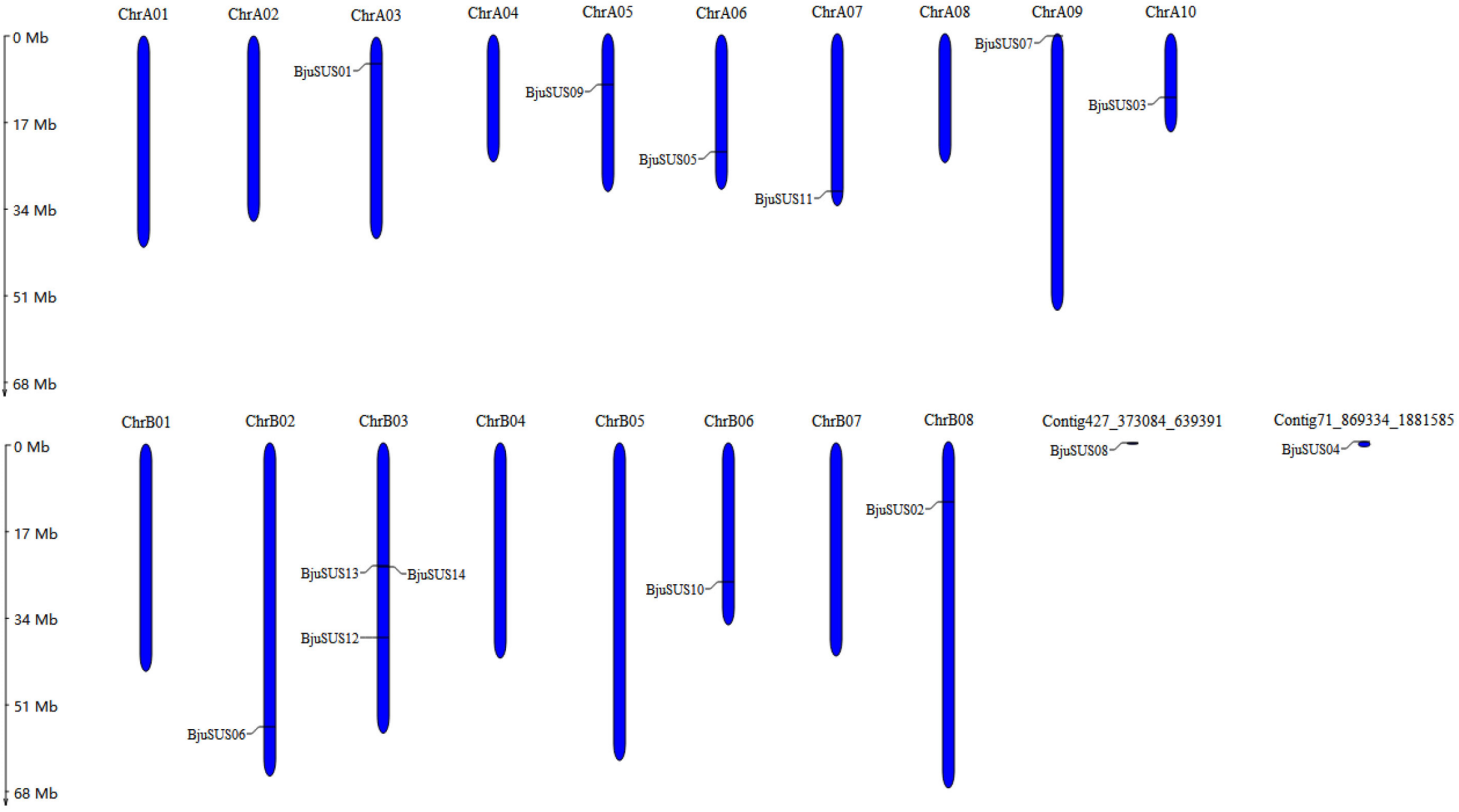
Chromosomal localization of the *BjuSUSs*. The chromosome number is shown at the top of each chromosome.

These 14 genes were widely dispersed on 10 of 18 *B. juncea* chromosomes and two contigs (Fig. 1). The number of chromosomal gene distribution was similar in sub-genomes A and B. The *BjuSUS13* and *BjuSUS14* genes were shown to be accumulated on the same region of the chromosome. This proves the clustering phenomenon. Three *BjuSUSs* (*BjuSUS12*, *13* and *14*) were located on the chrB03, while the other chromosomes only had one *BjuSUS* gene.

### Phylogenetic relationships of BjuSUS genes

**Figure 2:**
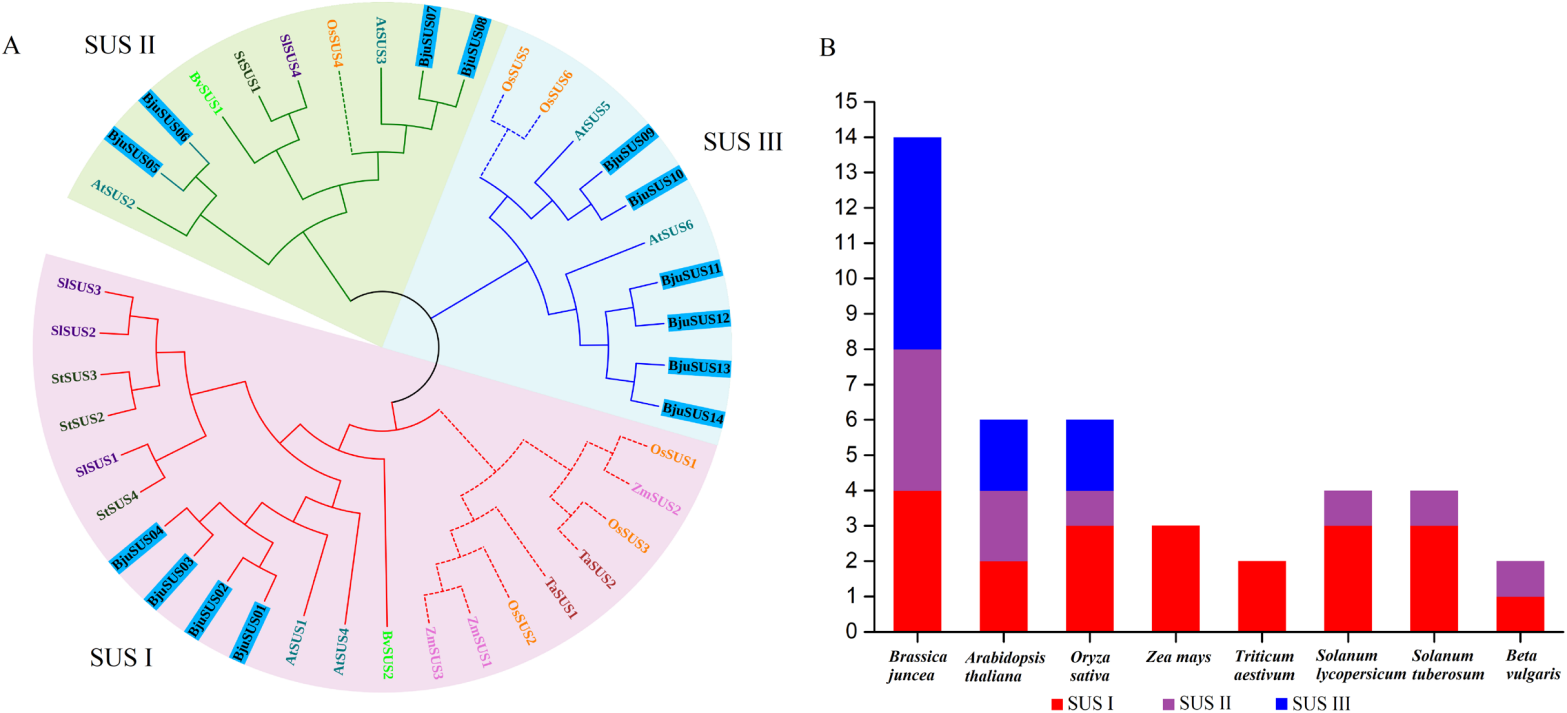
Phylogentic tree derived from amino acid sequences of the sucrose synthase genes in *B. juncea* (*BjuSUS*) and other seven species. (A) The phylogenetic tree was constructed using the neighbor-joining method with 1,000 bootstrap replicates. The branched lines of the subtrees are colored to indicate different SUS groups, the solid line branches represent dicots, and the dashed lines represent monocots. (B) SUS family members of *B. juncea* (*BjuSUS*), *A. thaliana* (*AtSUS*), *Oryza sativa* (*OsSUS*), *Zea mays* (*ZmSUS*), *Triticum aestivum* (*TaSUS*), *Solanum lycopersicum* (*SlSUS*), *Solanum tuberosum* (*StSUS*) and *Beta vulgaris* (*BvSUS*).

A phylogenetic tree of the mustard and eight other species of SUS proteins was constructed to determine the evolutionary relationships of the SUS gene family in *B. juncea*. As shown in Fig. 2A, the SUS genes were classified into three groups based on phylogenetic analysis: SUS I, SUS II and SUS III. The SUS I group was branched into the monocot and dicot subgroup while SUS II and SUS III groups were branched into the monocot subgroup only. Phylogenetic analysis revealed that the SUS family members of dicot and monocot plants were distributed in SUS I to SUS III. This implies that amplification of the SUS gene family occurs before differentiation of the dicot and monocot plants. Furthermore, all the SUSs in maize and *Triticum aestivum* occur in SUS I. Each SUS I and SUS II group contained four BjuSUSs while the SUS III family contained six BjuSUSs (Fig. 2B). The phylogenetic relationship diversity exhibited by the BjuSUSs show the discrete biological role for their paralogs, although multiple sequence alignments have proved that they have high sequence similarities.

### Structure and motif location analysis of BjuSUSs

**Figure 3:**
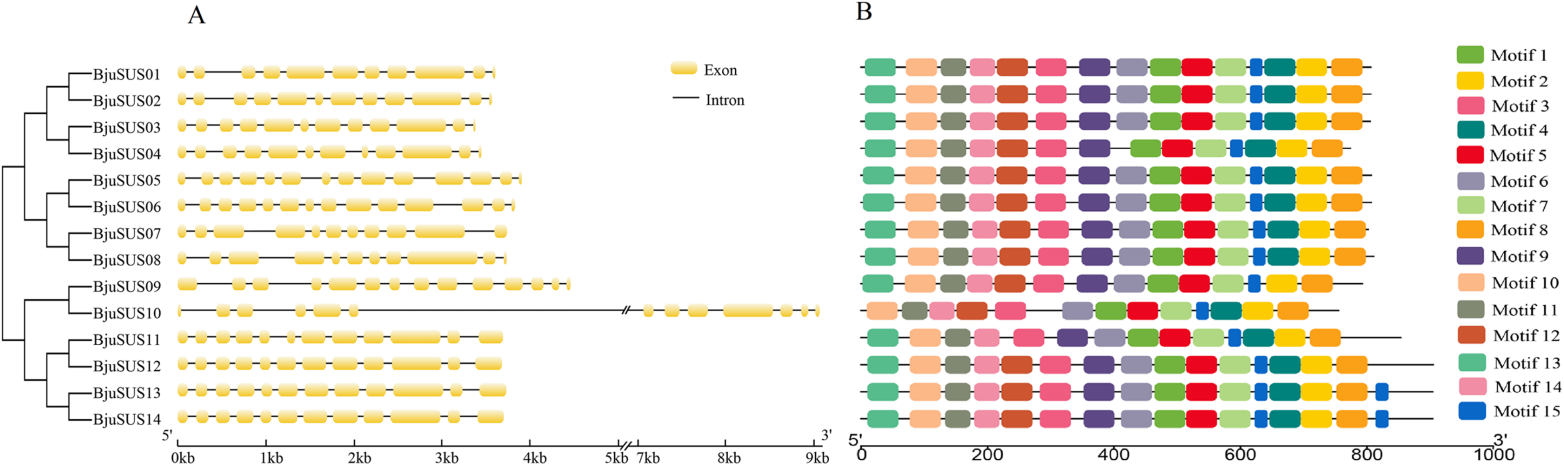
Schematic gene structure and motif location of 14 sucrose synthase genes in *B. juncea*. (A) Structure of *BjuSUSs*. Introns and exons are represented by black lines and yellow boxes, respectively. (B) Distribution of 15 conserved motifs of BjuSUS proteins. Each colored box represents conservative motif.

The exon/intron structure analysis showed that the 14 *BjuSUSs* contained multiple exons and introns that ranged from 11 to 14 and 10 to 13, respectively (Fig. 3A). The organization of the exons/introns was highly conserved, comparable to the high-level similarity observed by the alignment of the 14 amino acid sequences. The coding sequences of *BjuSUSs* were interspersed by the introns. The *BjuSUS10* gene had a long intron of approximately 5 kb sequence in length. It was the longest untranslated region.

Fifteen conserved motifs were finally identified from the BjuSUS proteins. The 14 members shared at least 12 common conserved motif compositions and exhibited a coincidental order of motif arrangement (Fig. 3B; Table S4). Motifs 6, 4 and 12 were missing in BjuSUS04, 09 and 11, respectively. In addition, the BjuSUS10 lacked motifs 13 and 9, while both BjuSUS13 and 14 genes had motif 15 at the end of the protein sequence. Motifs 1 and 5 were connected in each protein sequence, which was similar to motifs 2 and 4. However, the other motifs were separated.

### Evolutionary analysis of the BjuSUS genes

Type-I and type-II functional difference analyses were performed among the three SUS groups. As shown in Table 1, the type-I functional divergence coefficient θ_I_ was significantly greater than zero. It signified apparent type-I functional differences. This implied that amino acid site-specific selection may constraint the action in the different SUS groups and lead to specific evolution after diversification of different ethnic groups. The results from type-II functional divergence showed that the coefficient θ_II_ of any two groups was greater than zero. This finding implied that the proteins encoded by the three SUS subtypes exhibited significant type-II functional differentiation. The proteins encoded by the sub-type SUS genes have different physicochemical properties at certain amino acid positions, causing significant disproportionation. In the type-I analysis, 0, 3 and 0 critical amino acid sites (CAASs) that are associated with functional divergence were found in SUS I/SUS II, SUS I/SUS III and SUS II/SUS III groups, respectively. Moreover, in type-II analysis, the corresponding CAASs numbers were 59, 80 and 64, respectively. Variations in critical amino acid numbers between the type-II and type-I groups suggested the promotion of functional divergence in the evolution of the SUS family is mainly attributed to the site-specific shift of amino acid properties, followed by changes in the evolution rate.

**Table 1:** Functional divergence analysis of different groups of SUSs.

	Type-I				Type-II		
	θ_I_	SE	LRT	Qk > 0.9	θ_II_	SE	Qk > 0.9
SUS I/SUS II	0.3060	0.2957	0.5127	0	0.0798	0.0341	59
SUS I/SUS III	0.8060	0.2995	2.7033	3	0.0463	0.0462	80
SUS II/SUS III	0.3594	0.0671	19.1806	0	0.0169	0.0518	64

**Note:**

θ_I_, The coefficient of type-I functional divergence; θ_II_, The coefficient of type-II functional divergence; LRT, Likelihood ratio statisfics; *Q*_*k*_, Posterior probability.

The ratio of non-synonymous mutation frequency to synonymous mutation frequency for 15 homologous gene pairs in the SUS gene family was analyzed using the Ka/Ks calculation software. In the 15 paralogous gene pairs, all the Ka/Ks ratios were lower than 1, indicating that the 15 paralogous gene pairs were under a strong purifying selection during evolution (Table 2). Since deleterious mutations are usually eliminated by the purification selection process, SUS genes may share conserved functions in *B. juncea* species.

**Table 2:** Ka, Ks and Ka/Ks of 15 paralogous gene pairs of BjuSUSs. These values are based on full-length CDS of 14 gene pairs of BjuSUSs.

Paralogous gene pair	Ka	Ks	Ka/Ks
BjuSUS01/BjuSUS02	0.0070	0.2249	0.0312
BjuSUS01/BjuSUS03	0.0141	0.3234	0.0436
BjuSUS01/BjuSUS04	0.0113	0.3198	0.0353
BjuSUS02/BjuSUS03	0.0169	0.2753	0.0612
BjuSUS02/BjuSUS04	0.0130	0.2467	0.0527
BjuSUS03/BjuSUS04	0.0068	0.1704	0.0396
BjuSUS05/BjuSUS06	0.0122	0.1999	0.0612
BjuSUS07/BjuSUS08	0.0161	0.2136	0.0755
BjuSUS09/BjuSUS10	0.0042	0.0472	0.0900
BjuSUS11/BjuSUS12	0.0228	0.2091	0.1091
BjuSUS11/BjuSUS13	0.0371	0.2831	0.1310
BjuSUS11/BjuSUS14	0.0353	0.3182	0.1109
BjuSUS12/BjuSUS13	0.0419	0.2829	0.1482
BjuSUS12/BjuSUS14	0.0389	0.3053	0.1273
BjuSUS13/BjuSUS14	0.0101	0.1651	0.0610

### Synteny analysis of the SUS gene in *B. juncea* and Arabidopsis

**Figure 4:**
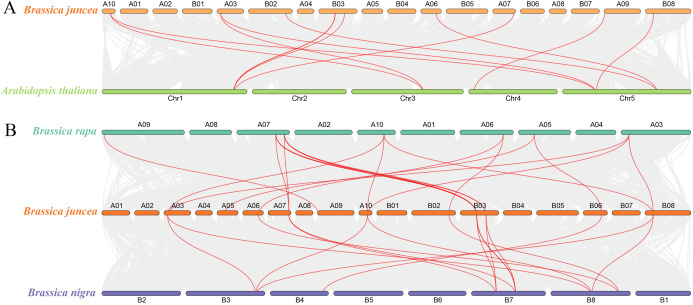
Analysis of collinearity among cruciferous species. (A) Analysis of collinearity between *B. juncea* and *A. thaliana* genomes. (B) Analysis of collinearity among *B. juncea*, *Brassica rapa* and *Brassica nigra* genomes. Colored circular rectangles denote the chromosomes of two plants, while bezier curves connect protein-coding genes in the synteny blocks. The red curves represent gene pairs that are collinear with some *BjuSUS* genes.

**Figure 5:**
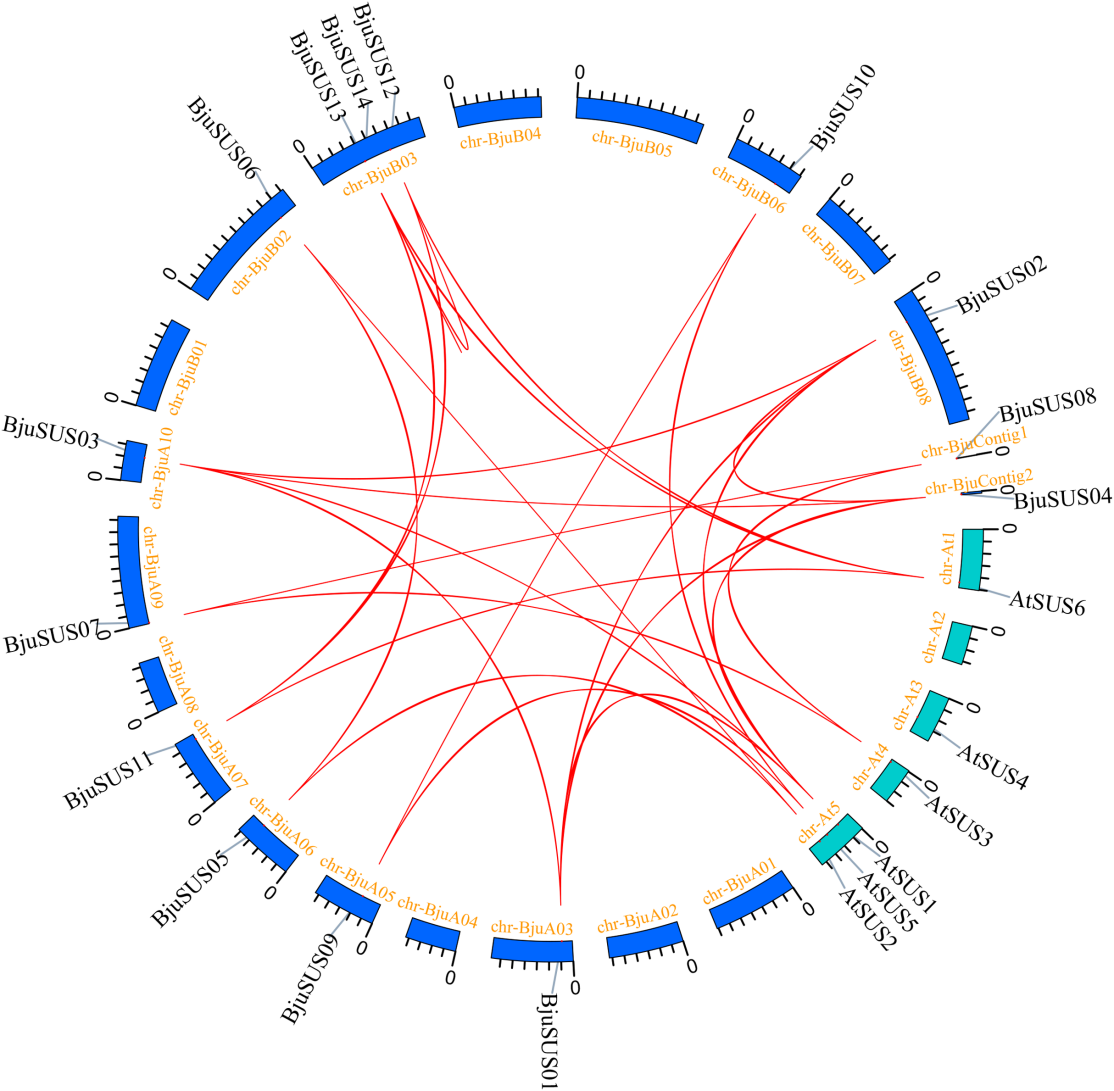
Segmental duplication and synteny analysis of SUS gene in *B. juncea* and *A. thaliana*. The chromosomes of the two species are shown as circular in different colors. Each SUS gene is marked with a short line on the chromosome, and the red curve indicates a collinear gene pair.

To determine the evolutionary relationship and degree of homology between mustard and *Arabidopsis*, their comparative synteny at the genome level was analyzed to elucidate the origin of the *BjuSUSs* (Fig. 4A). There were 2,208 synteny blocks between the *B. juncea* and *Arabidopsis* genomes, of which 66.71% were collinear. In the largest linear region, 648 gene pairs were shared between the *B. juncea* chrA02 and *Arabidopsis* chr5. This implied that mustard and *Arabidopsis* have a high homology. Collinearity analysis of *AtSUSs* and *BjuSUSs* was used to identify homologous candidate genes for important traits such as morphological development, yield, quality and metabolism of the mustard (Fig. 5). Collinearity analysis revealed 29 SUS gene pairs in *B. juncea* and *Arabidopsis*. Among them, fifteen *BjuSUSs* pairs showed fragmental duplication. These findings revealed that most SUS genes are orthologous in both species. The results show that some *BjuSUSs* are co-linear with *AtSUSs*. *BjuSUS01*, *02*, *03* and *04* were found to be homologous to *AtSUS1*.

The evolutionary relationships of BjuSUS genes were further determined based on the genetic relationships between the *Brassica* crops (Fig. 4B). In the collinearity analysis of *B. juncea*, *Brassica rapa* and *Brassica nigra*, it was found that the synteny blocks between *B. juncea* and *B. rapa* was more than 70%. The same finding was established between *B. juncea* and *B. nigra*. The higher homology revealed that *B. juncea* was the offspring of *B. rapa* and *B. nigra*. Twelve *BjuSUSs* were collinear with the genes in *B. rapa* while 10 *BjuSUSs* were collinear with the genes in *B. nigra*. Most of the *BjuSUS* genes were homologous to the two genes in *B. rapa* and *B. nigra*, respectively.

### Analysis of cis-regulatory elements and functional annotation of BjuSUS genes

**Figure 6:**
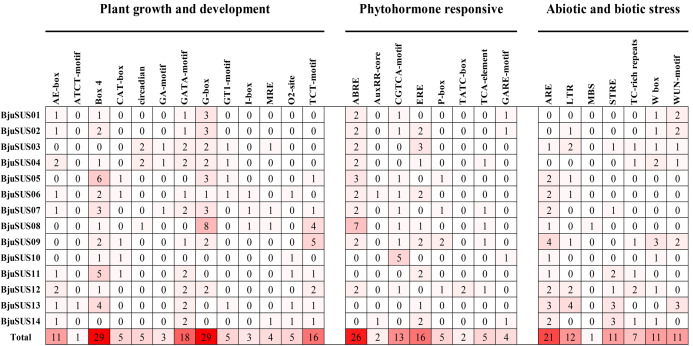
Analysis of *cis*-regulatory elements in the promoter regions of mustard SUS genes. The figures indicate the number of each cis-acting element in the promoter region (1.5 kb upstream of the translation start site) of *BjuSUSs*. Based on the functional annotation, the *cis*-acting elements were classified into three major classes: plant growth and development, phytohormone responsive, or abiotic and biotic stresses-related *cis* elements. Analysis of *cis*-regulating elements in the promoter region of mustard gene.

All *cis*-regulatory elements in the promoter region of *BjuSUSs* were analyzed using the Plant-CARE database. Elements were classified into three groups based on their functional associations; plant growth and development (13 elements), phytohormone response (8 elements) as well as abiotic and biotic stress (7 elements) (Fig. 6). The results showed that the light-responsive elements (Box-4, G-box, GATA-motif and TCT-motif) appeared 29, 29, 18 and 16 times in the 14 *BjuSUSs*, respectively. These four *cis*-regulatory elements were widely distributed in the *BjuSUSs* promoter region and may be involved in the regulation of mustard growth and development. In particular, G-box appeared eight times in *BjuSUS08* while Box 4 appeared six times in *BjuSUS05*, indicating that they may be significant in light responsiveness and photosynthetic product accumulation. Moreover, 26 abscisic acid responsive elements (ABRE) and 21 anaerobic induction elements (ARE) were detected in *BjuSUSs* promoters.

GO annotation was performed to predict the functions of 14 BjuSUS genes. A total of 116 annotation results were obtained and further divided into 23 GO terms, which were in turn assigned into three ontology categories: biological process (BP), cellular component (CC), and molecular function (MF) (Table S5). Biological process made up the majority of the GO annotations, followed by cellular component and molecular function. Among the annotations, all the BjuSUSs were involved in “sucrose metabolic process” and “sucrose synthase activity”.

### Expression patterns of BjuSUS genes

#### Expression levels of BjuSUS genes at different stem developmental stages

**Figure 7:**
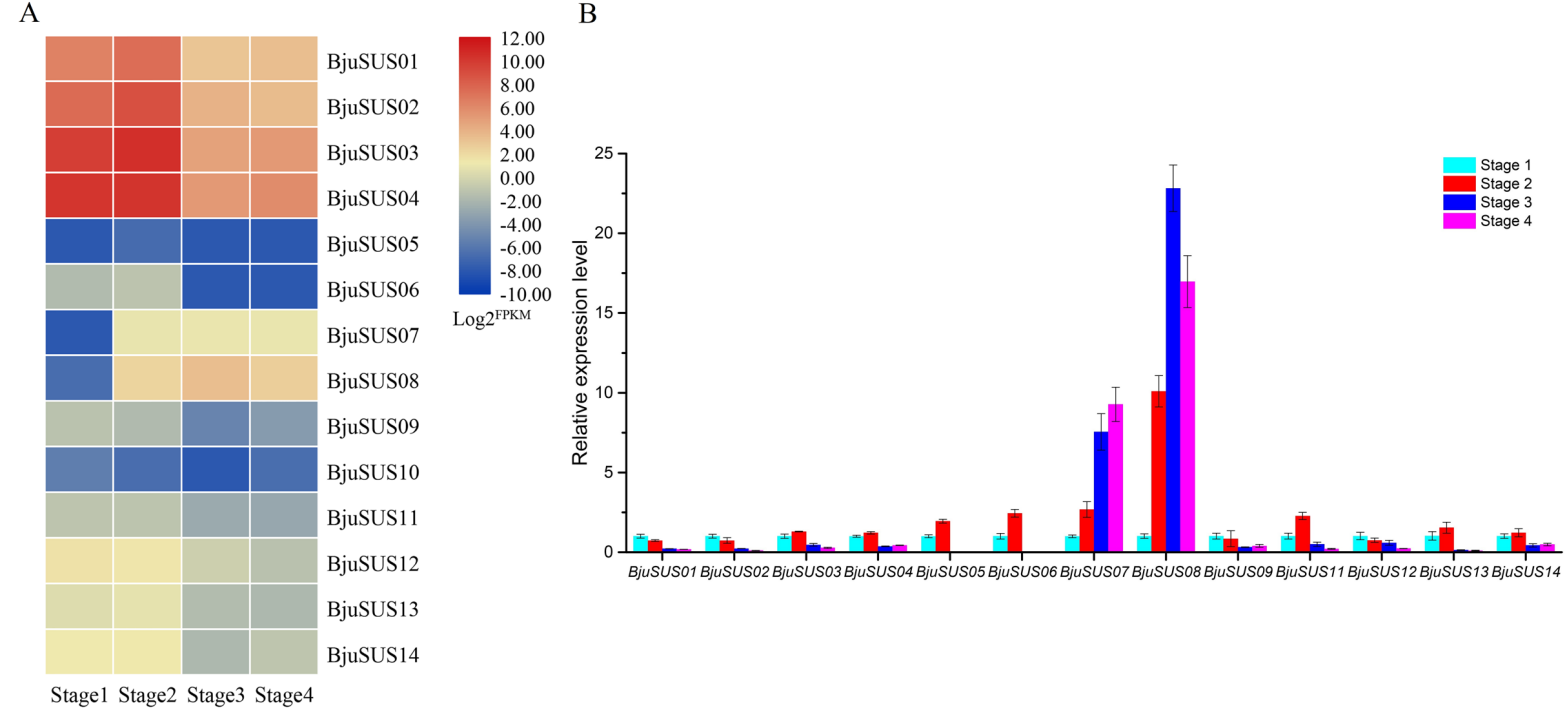
Expression analysis of *BjuSUSs* at different stem developmental stages. (A) Expression patterns of SUS family genes were analyzed based on transcriptome data. (B) Expression levels of the *BjuSUSs* from different stages of stem swelling of mustard.

Analysis of the expression abundance of *BjuSUSs* in the transcriptome data at different developmental stages revealed that the expression of each *BjuSUS* gene has similarities and differences (Fig. 7A). Among them, *BjuSUS01*-*04* maintained a high transcription level at each stage, especially in the first two stages. Its expression levels were highest in the second stage, with a significant decrease in the last two developmental stages. In addition, the expression of *BjuSUS07* and *08* was low in the first stage, but was significantly elevated in the latter stage. *BjuSUS05*, *06* and *10* showed relatively low expression profiles throughout the stem development stages. As shown in Fig. 7B, most *BjuSUSs* exhibited a distinct but partially overlapping expression trend, that is, high expression in the early stages of stem swelling, and low expression levels in the latter stages of development. However, there were no detectable expression levels in the latter two stages of *BjuSUS05* and *06*. *BjuSUS07* and *08* were highly expressed in the latter stages of development. These findings were consistent with the results of the transcriptome analysis.

#### Expression patterns of *BjuSUS* genes in response to phytohormones

**Figure 8:**
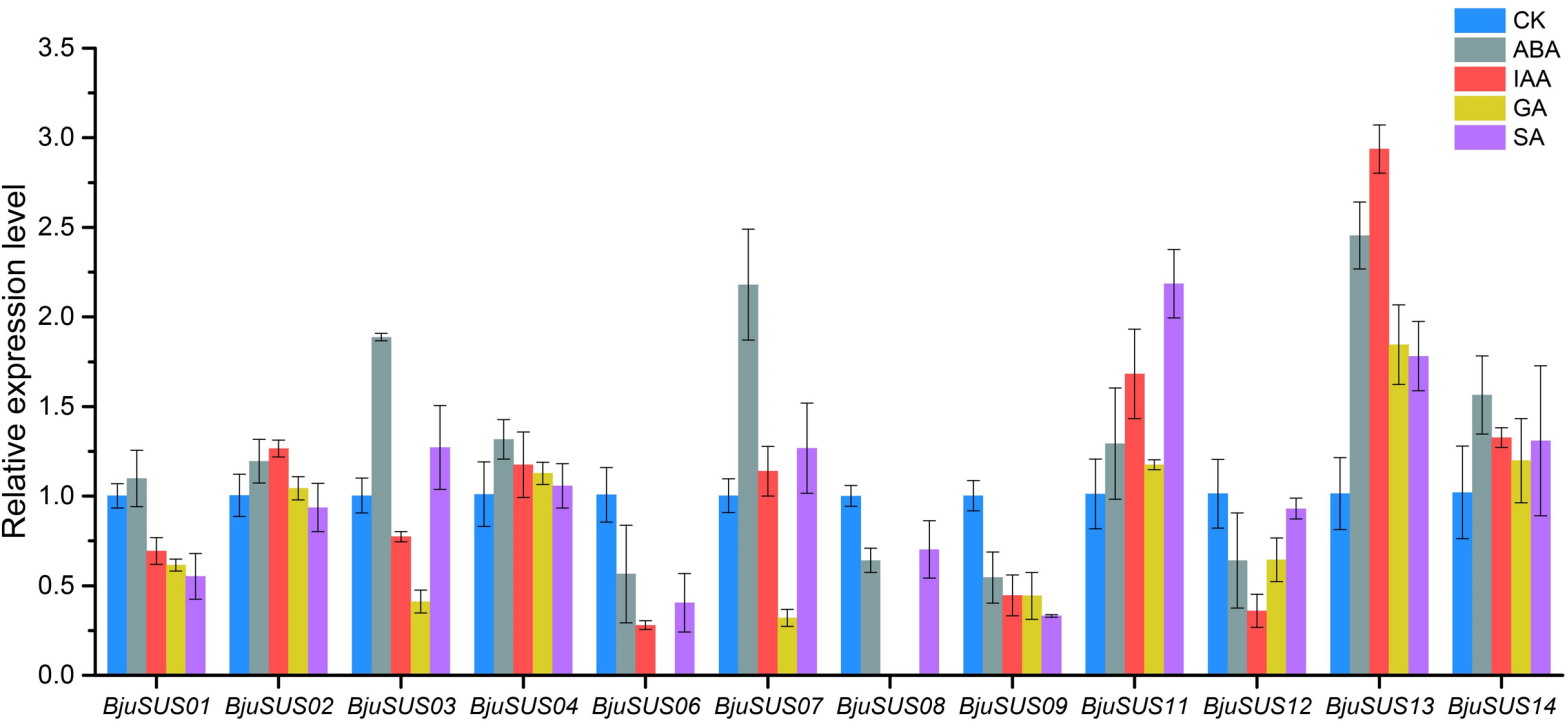
Expression profiles of *BjuSUSs* in response to treatment with abscisic acid (ABA), auxin (IAA), gibberellin (GA) and salicylic acid (SA).

The expression patterns of *BjuSUSs* in response to abscisic acid (ABA), auxin (IAA), gibberellin (GA) and salicylic acid (SA) were determined (Fig. 8). The expression levels of *BjuSUS04*, *11*, *13* and *14* were up-regulated after treatment with four phytohormones. The expression levels of *BjuSUS13* were significantly elevated. In addition, most genes, especially *BjuSUS03* and *07*, exhibited elevated expression levels after ABA treatment. *BjuSUS02* and *07* exhibited up-regulated expression levels after IAA treatment, while the expression levels of *BjuSUS03* and *07* were also up-regulated after SA treatment. Furthermore, the *BjuSUS06*, *08*, *09* and *12* were all down-regulated with hormones.

#### Expression patterns of *BjuSUS* genes in response to abiotic stresses

**Figure 9:**
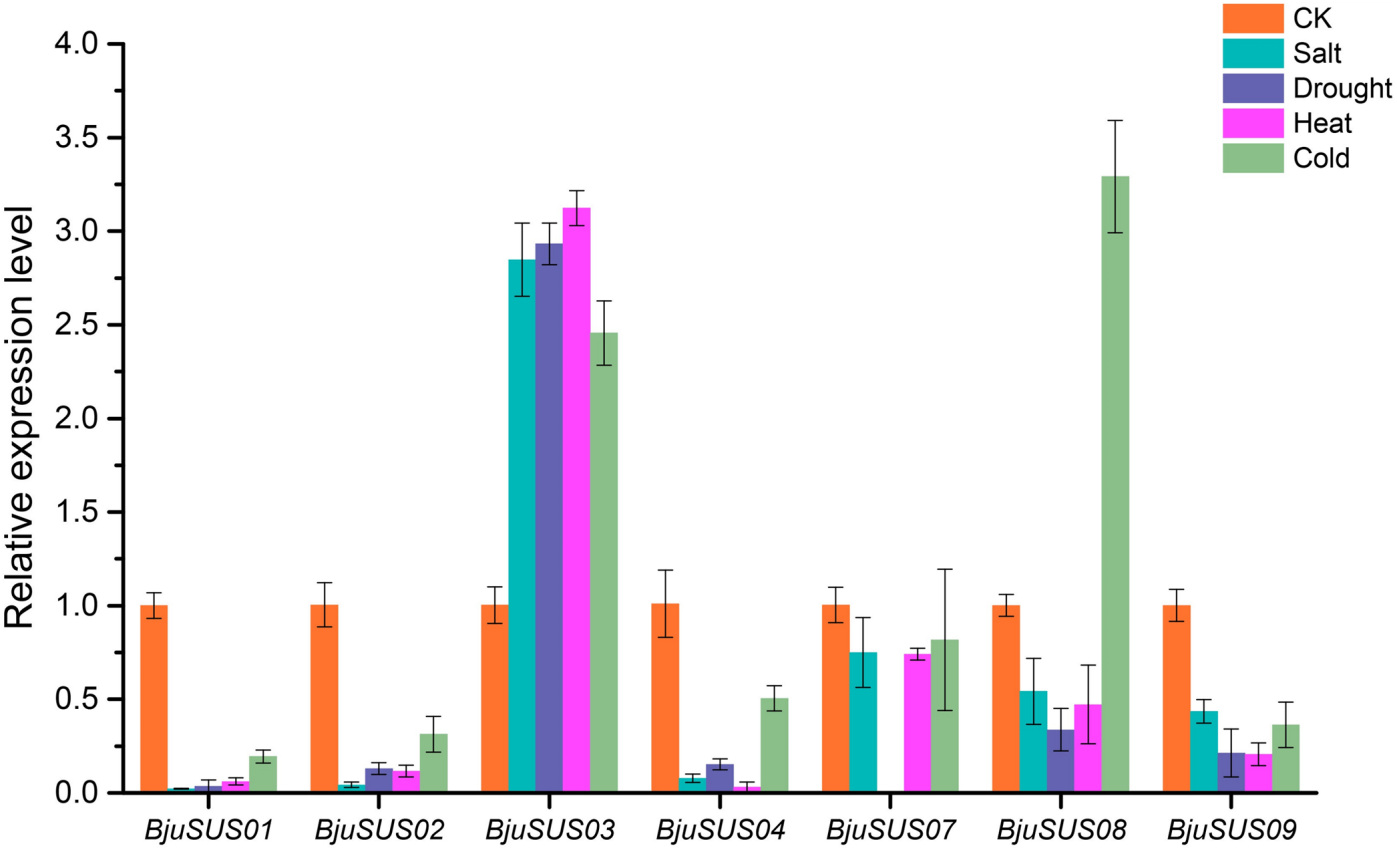
Expression profiles of *BjuSUSs* in response to treatment with salt, drought, heat temperature and cold temperature stress.

To determine whether *BjuSUSs* are involved in abiotic stress, qRT-PCR was used to measure their expression levels under salt, drought, and high and low temperature stress. As shown in Fig. 9, the expression level of most *BjuSUSs* decreased under stress treatment compared to the control group, however, the expression patterns were different when subjected to different stresses. Most of the genes were down-regulated, with some expressions not being detected. The expression levels of *BjuSUS03* were significantly up-regulated under salt, drought heat and cold stress, while the expression levels of *BjuSUS08* were significantly up-regulated under cold stress. In response to abiotic stress, *BjuSUS01* and *02* exhibited similar expression patterns.

## Discussion

Comparative genomics approaches have been used to analyze SUS gene families in various plant species such as *Arabidopsis*, rice, maize, cotton, grape, peach and poplar (Xu et al., 2019). In this study, 14 SUS genes from *B. juncea* were identified. Based on their molecular structures, evolutionary models and expression profiles, the *BjuSUSs* play an important role in the development of *B. juncea* stems.

### Evolutionary conservation among BjuSUS genes

Phylogenetic, structural and evolutionary pattern analyses are fundamental in predicting the possible genetic and evolutionary relationships as well as potential functions of gene families. Phylogenetic tree analysis showed that the mustard SUS genes were associated with dicotyledonous plants such as *Arabidopsis*. The *BjuSUSs* were found to be closely clustered in the subgroups. This emphasizes the diversity of SUS genes in monocot and dicot plants. Studies have documented that the protein structure, the length and positional characteristics of introns and exons of the SUS family genes are highly conserved among different species. This protein structure provides important information regarding evolutionary relationships between genes (Lecharny et al., 2003). We found that *BjuSUSs* have 10–13 introns, and most of the exons between SUS I, SUS II and SUS III groups are highly similar, which slightly differs from the 11–13 introns in the peach (Zhang et al., 2015). In addition, the number of introns in *PtrSUS* in the *Populus* and *MdSUS* in the apple is between 10 and 14 (Tong et al., 2018; Zhang et al., 2011). These results indicate that the number of introns in the SUS gene family among different species is highly conserved.

To determine the evolutionary relationships of the *BjuSUS* gene family and explore their possible functions, gene duplication events and homology analysis were performed. Tandem duplications (*BjuSUS13* and *BjuSUS14*) were found in chromosomal mapping. Collinearity analysis of mustard and *Arabidopsis* revealed that there were more fragment duplications between *BjuSUSs*. Duplicated gene pairs were all from the same subgroup, with highly similar exon/intron structures and protein characteristics. This implies that the amplification methods of mustard SUS gene family include tandem and fragment duplication, which may increase copy numbers in the gene family, resulting in functional redundancy (Cannon et al., 2004). Furthermore, the Ka/Ks results showed that during evolution, *BjuSUSs* eliminated harmful mutations through purification and selection in order to maintain a conservative structure and functions. Homological analysis provides a basis for the evolutionary and functional characterization of the SUS gene family in mustard and *Arabidopsis*. Homology analysis revealed that 14 *BjuSUSs* exhibited a homology with 5 *AtSUSs* in *Arabidopsis*. The *BjuSUS* genes and their corresponding *AtSUS* genes have a close phylogenetic relationship, which strongly implies a certain degree of functional similarity. *Brassica* and *Arabidopsis* belong to the cruciferous family, but diverged about 20 million years ago (Vision, Brown & Tanksley, 2000). After being separated from the *Arabidopsis*, the *Brassica* species such as *B. rapa* and mustard have tripled at the genome level. Moreover, in the “U-triangle” theory, there are three basic subgenomes (A subgenome, B subgenome and C subgenome), and *B. juncea* (AABB, 2*n* = 36) is a heterotetraploid formed by interspecific hybridization of *B. rapa* (AA, 2*n* = 20) and *B. nigra* (BB, 2*n* = 16) (Nagaharu, 1935). Chromosomal rearrangements, translocations, duplications, and gene deletions during doubling greatly promoted the evolution of *Brassica*. Some SUS genes in mustard have paralogs, and they all exhibited a homology with certain genes (*BjuSUS05/06* and *BjuSUS01/02/03*) in *B. rapa* and *B. nigra*. These findings confirm an evolutionary relationship. The mustard did not undergo a larger chromosomal doubling process after the triploid event (Song et al., 2020). However, some *BjuSUS* genes did not find their corresponding genes, indicating that the SUS family members may have been lost or mutated to varying degrees during evolution.

### Divergence in BjuSUS gene expression patterns

#### *BjuSUS* genes involved in mustard growth and development

Plant growth and development is a complex process. A majority of the *cis*-regulatory elements associated with plant growth and development are found in the promoter regions. In this study, four genes (*BjuSUS01*, *02*, *03* and *04*) were highly expressed during growth and development. The expression levels exhibited an overall decreasing trend, implying that these genes are more important in the early stages of stem development. However, their high expression levels in stems indicate that they are involved in the entire developmental stage of the stem and sucrose metabolism. Similar results have also been reported in other studies. Overexpressing the bamboo *BeSUS5* gene increased the stem cellulose content, cell wall thickness and fiber quality in transgenic poplar (Huang et al., 2020). Also, the *TcSUS3* gene in cacao pods exhibited a downward expression trend in the developmental stage of the pods (Li et al., 2015). Similar findings were observed in the expression of the *SBSS2* gene in sugarbeet root tissues (Haagenson, Klotz & McGrath, 2006).

The four *BjuSUSs* were found to be structurally similar and belonged to the SUS I group. This implied that they exhibit the same functions during stem development. Studies have shown that the transcription level of the *AtSUS1* gene in *A. thaliana* is highest in the stem while the expression levels of *OsSUS1*, *OsSUS2* and *OsSUS3* genes in rice are highest during the early caryopsis development stages (Bieniawska et al., 2007; Hirose, Scofield & Terao, 2008). These genes belong to the SUS I group and exhibit a high level of redundancy, where some genes with similar expression patterns have similar functions. The six *AtSUS* genes also exhibit distinct but partially overlapping expression patterns (Baud, Vaultier & Rochat, 2004). Except for *BjuSUS01-04*, the expression levels of *BjuSUS* genes was low or not expressed, while *BjuSUS07* and *08* were shown to be significantly up-regulated in the middle and late stages of stem swelling. During cucumber fruit development, the expression of *CsSUS4* in the latter stages has been shown to be significantly high when compared to the early stages. This indicates that they may play an important role in mustard development, thereby, affecting the size and weight of the stem (Fan et al., 2019). Understanding the structure, phylogeny, and homology of the SUS gene is essential in the functional analysis of the SUS gene family. Nucleotide and amino acid sequence similarities of the four *BjuSUSs* were all found to be above 90%, indicating that they have a high sequence homology. This finding lays credence to their structural and functional similarities.

#### Phytohormones induce *BjuSUSs* expression in mustard

When subjected to phytohormonal pressure, the expression levels of some genes in the mustard SUS family were altered. After being subjected to ABA, the expression levels of most genes were shown to be up-regulated. It has been previously reported that ABA elevates the expression levels of grape SUS gene in grape berry calli (Wang et al., 2017). *BjuSUSs* are up-regulated when exposed to GA while *GhSusA1* was shown to be upregulated in fiber culture after exogenous administration of gibberellin (Bai et al., 2014). Furthermore, the expression levels of *BjuSUS03*, *07*, *11* and *13* after phytohormonal treatment were found to be significantly different from those of the control group. Therefore, we hypothesized that *BjuSUSs* are involved in responses to a variety of phytohormone signals and regulate the associated physiological processes.

#### Abiotic stress-induced *BjuSUSs* expression in mustard

During plant growth and development, many abiotic stresses are encountered. The SUS gene family has been shown to be involved in regulating stress responses. For example, it can induce *AtSS1* gene expression in *A. thaliana* when the plant is exposed to cold, drought or hypoxic stress factors (Baud, Vaultier & Rochat, 2004). Members of the mustard SUS gene family showed different expression patterns in leaves when exposed to different abiotic stresses. When exposed to salt, drought, heat, and cold stress, the expression levels of *BjuSUS03* were significantly up-regulated, indicating that abiotic stress induces its overexpression to enhance the ability of the mustard to resist stress. The overexpression of *OsSUS3*, which belongs to the SUS I group, suppresses the damage associated with high temperatures to rice yield and quality (Takehara et al., 2018). Furthermore, the expression of *BjuSUS08* was found to be significantly up-regulated in cold stress conditions and down-regulated in the presence of other abiotic stressors. The expression level of tobacco *Sus5* was also shown to be significantly elevated after low-temperature treatment (Wang et al., 2015). The expression levels of the other *BjuSUSs* were significantly down-regulated after exposure to the different abiotic stress factors. These findings are in tandem with those documented in grapes (Zhu et al., 2017) and rubber trees (Xiao et al., 2014).

## Conclusions

Fourteen SUS genes were identified in mustard. Collinearity analysis revealed that the SUS gene family may be lost or mutated to varying degrees after genome doubling or hybridization of *B. rapa* and *B. nigra t*o form mustard. The expression patterns of *BjuSUSs* in different stages of mustard stem development indicate that *BjuSUS01-04* have an important role in the biological processes associated with development. Plant hormones and abiotic stress induced the expression of *BjuSUSs*, indicating that this gene family is involved in their regulation.

## Supplemental Information

10.7717/peerj.10878/supp-1 Supplemental Information 1 Primer sequences designed for *BjuSUS* genes.

10.7717/peerj.10878/supp-2 Supplemental Information 2 Basic information of SUS gene family members in *B. juncea*.

10.7717/peerj.10878/supp-3 Supplemental Information 3 Amion acid sequence pairwise comparisons (% similarity) between sucrose synthase genes in *B. juncea*.

10.7717/peerj.10878/supp-4 Supplemental Information 4 Putative conserved motifs information of BjuSUSs.

10.7717/peerj.10878/supp-5 Supplemental Information 5 Sequence and GO annotation information of SUS gene family members in *B. juncea*.

10.7717/peerj.10878/supp-6 Supplemental Information 6 Raw data. SUS proteins used in phylogentic tree were represented in Sheet 1. Raw data corresponding to Figs. 7, 8 and 9 were represented in Sheet 2.

## Additional Information and Declarations

### Data Availability

The following information was supplied regarding data availability: RNA-Seq data are available at the NCBI Sequence Read Archive: SRP151320 The genome sequence data of *Brassica juncea* are also available at NCBI: PRJNA285130 Raw data are available as a Supplemental File.
