# Supplementary material for: Sucrose synthase gene family in Brassica juncea: genomic organization, evolutionary comparisons, and expression regulation
Source: PeerJ. 2021 Mar 9;9:e10878. doi: 10.7717/peerj.10878 (PMC7953879; doi:10.7717/peerj.10878)
Supplement: Supplemental Information 1 [file peerj-09-10878-s001.docx]

**Table S1:**

**Primer sequences designed for *BjuSUS* genes.**

| **Gene ID** | **Forward primer (5'→3')** | **Reverse primer (5'→3')** |
| --- | --- | --- |
| *BjuSUS01* | CGGCAAGTCAGGCTTCCACATT | TCAAGGTAACGGCGGCTCTCA |
| *BjuSUS02* | GTTCGTGCCTTGGAGACAGAGA | CGCAGCATCCTCGGTGTAAGT |
| *BjuSUS03* | GGCGGCTGATACTCTTGCTGAT | CTCAAGGTAACGACGGCTCTCA |
| *BjuSUS04* | ACCTCTCGGCTAAGCTCTTCCA | TCAAGGACACGCTCGGCATTG |
| *BjuSUS05* | TCCTCGTCCAACTCGGTCATCA | ACTCCTCTGCCCTCGCCAAA |
| *BjuSUS06* | CTCAATCGCCACCTCTCGTCAA | AACCTTCTGTGCCGTGTCACC |
| *BjuSUS07* | TGTCAATGTCTACGAGCTAAGCGT | CTGCTGCTAGCTCCATCAACGAG |
| *BjuSUS08* | GGCTCACTAGGATTCTAAGCACGA | CAGCTCGTAGACGTTAACGCGAA |
| *BjuSUS09* | TTATACCCATAGTGAGTGATATTCC | CGAGCCTCGAGTTGCTGATGG |
| *BjuSUS10* | CACTTATAGCTATTGGAGGCATTTG | CTTTGAGCTTCTAGTTGCTGATGAT |
| *BjuSUS11* | GATCGGATTCTATCGCAGACAA | AAGCCGAGGAGTTGTGAAGTCA |
| *BjuSUS12* | CGCCGACCAATCTGTGTACTTC | GGTTCTGTTGTAATCTGCATGG |
| *BjuSUS13* | CCAACAACACTTCCACGATCCG | ACCACTGCTGCCTCCTGAGT |
| *BjuSUS14* | CGATGCAAAGTGGAAAGAACTAGAC | GGTATCCCATGTGTTCATTATTG |
| *UBC* | CGTCTGCTTAGTGAACCTGCTC | AAGGAGACTGTGTAGGACCAAGAA |
