# Supplementary material for: Sucrose synthase gene family in Brassica juncea: genomic organization, evolutionary comparisons, and expression regulation
Source: PeerJ. 2021 Mar 9;9:e10878. doi: 10.7717/peerj.10878 (PMC7953879; doi:10.7717/peerj.10878)
Supplement: Supplemental Information 2 [file peerj-09-10878-s002.docx]

**Table S2:**

**Basic information of SUS gene family members in *B. juncea*.**

| Name | Gene ID | Genomic position | ORF (bp) | Exon number | No. of amino acids | Molecular weight (kDa) | Theoretical *pI* | Subcellular localization |
| --- | --- | --- | --- | --- | --- | --- | --- | --- |
| BjuSUS01 | BjuA009339 | A03:5454469..5458075 | 3607 | 11 | 806 | 92.48 | 5.67 | cyto/ M |
| BjuSUS02 | BjuB015313 | B08:12249086..12252654 | 3569 | 12 | 806 | 92.40 | 5.90 | cyto/M |
| BjuSUS03 | BjuA047153 | A10:12904806..12908187 | 3382 | 12 | 805 | 92.25 | 5.80 | cyto/M |
| BjuSUS04 | BjuO008945 | Contig71_869334_1881585:757950..761396 | 3447 | 12 | 774 | 88.54 | 5.83 | cyto/M |
| BjuSUS05 | BjuA023848 | A06:23606873..23610780 | 3908 | 14 | 807 | 91.95 | 5.89 | cyto/_ |
| BjuSUS06 | BjuB037515 | B02:57413398..57417223 | 3826 | 14 | 807 | 92.02 | 5.86 | chlo/_ |
| BjuSUS07 | BjuA036504 | A09:526131..529867 | 3737 | 11 | 802 | 91.39 | 5.99 | mito/M |
| BjuSUS08 | BjuO006586 | Contig427_373084_639391:183607..187340 | 3734 | 11 | 811 | 92.51 | 5.93 | mito/M |
| BjuSUS09 | BjuA018844 | A05:10253196..10257656 | 4461 | 14 | 793 | 90.12 | 6.20 | cyto/_ |
| BjuSUS10 | BjuB022852 | B06:28028348..28037415 | 9068 | 13 | 755 | 85.42 | 5.82 | cyto/_ |
| BjuSUS11 | BjuA043452 | A07:31843382..31847074 | 3693 | 12 | 854 | 97.09 | 6.00 | cyto/_ |
| BjuSUS12 | BjuB030220 | B03:39367349..39371030 | 3682 | 12 | 905 | 102.93 | 6.21 | cyto/_ |
| BjuSUS13 | BjuB030962 | B03:24899338..24903068 | 3731 | 12 | 904 | 102.47 | 6.36 | cyto/C |
| BjuSUS14 | BjuB047347 | B03:25128525..25132226 | 3702 | 12 | 904 | 102.48 | 6.17 | cyto/_ |

Cyto: cytoplasm; mito: mitochondrion; chlo: chloroplast; M: mitochondrion; C: chloroplast; -: any other location.
