# Supplementary material for: Sucrose synthase gene family in Brassica juncea: genomic organization, evolutionary comparisons, and expression regulation
Source: PeerJ. 2021 Mar 9;9:e10878. doi: 10.7717/peerj.10878 (PMC7953879; doi:10.7717/peerj.10878)
Supplement: Supplemental Information 3 [file peerj-09-10878-s003.docx]

Table S3:

Amion acid sequence pairwise comparisons (% similarity) between sucrose synthase genes in *B. juncea*.

|  | BjuSUS01 | BjuSUS02 | BjuSUS03 | BjuSUS04 | BjuSUS05 | BjuSUS06 | BjuSUS07 | BjuSUS08 | BjuSUS09 | BjuSUS10 | BjuSUS11 | BjuSUS12 | BjuSUS13 | BjuSUS14 |
| --- | --- | --- | --- | --- | --- | --- | --- | --- | --- | --- | --- | --- | --- | --- |
| BjuSUS01 | - |  |  |  |  |  |  |  |  |  |  |  |  |  |
| BjuSUS02 | 98.64 | - |  |  |  |  |  |  |  |  |  |  |  |  |
| BjuSUS03 | 97.27 | 96.77 | - |  |  |  |  |  |  |  |  |  |  |  |
| BjuSUS04 | 97.55 | 97.16 | 98.58 | - |  |  |  |  |  |  |  |  |  |  |
| BjuSUS05 | 66.96 | 66.83 | 66.67 | 66.75 | - |  |  |  |  |  |  |  |  |  |
| BjuSUS06 | 66.96 | 66.83 | 66.67 | 66.36 | 97.52 | - |  |  |  |  |  |  |  |  |
| BjuSUS07 | 68.47 | 68.34 | 68.47 | 67.84 | 74.78 | 75.41 | - |  |  |  |  |  |  |  |
| BjuSUS08 | 67.83 | 67.58 | 67.54 | 67.01 | 73.70 | 74.57 | 97.01 | - |  |  |  |  |  |  |
| BjuSUS09 | 53.32 | 53.46 | 53.00 | 52.08 | 52.65 | 52.65 | 54.68 | 54.29 | - |  |  |  |  |  |
| BjuSUS10 | 57.38 | 57.66 | 57.04 | 56.41 | 56.73 | 56.73 | 59.13 | 58.95 | 99.02 | - |  |  |  |  |
| BjuSUS11 | 56.99 | 57.12 | 57.07 | 56.06 | 55.60 | 55.60 | 56.73 | 56.09 | 65.91 | 69.17 | - |  |  |  |
| BjuSUS12 | 55.16 | 55.28 | 55.35 | 54.33 | 54.40 | 54.15 | 56.43 | 55.68 | 64.94 | 68.08 | 95.42 | - |  |  |
| BjuSUS13 | 55.40 | 55.53 | 55.35 | 54.33 | 54.09 | 54.34 | 56.05 | 55.56 | 65.15 | 68.57 | 92.50 | 92.23 | - |  |
| BjuSUS14 | 55.16 | 55.28 | 55.22 | 54.20 | 54.71 | 54.59 | 56.55 | 55.93 | 65.03 | 68.44 | 93.32 | 93.12 | 97.90 | - |
