# Supplementary material for: Sucrose synthase gene family in Brassica juncea: genomic organization, evolutionary comparisons, and expression regulation
Source: PeerJ. 2021 Mar 9;9:e10878. doi: 10.7717/peerj.10878 (PMC7953879; doi:10.7717/peerj.10878)
Supplement: Supplemental Information 4 [file peerj-09-10878-s004.docx]

**Table S4:**

**Putative conserved motifs information of BjuSUSs.**

| Name | E-Value | Width | Best Possible Match |
| --- | --- | --- | --- |
| Motif 1 | 4.2e-546 | 50 | YHFSCQFTADLIAMNNTDFIITSTYQEIAGSKERVGQYESHTAFTLPGLY |
| Motif 2 | 2.4e-465 | 50 | PTFATNQGGPAEIIVDGVSGFHIDPYHGDZAADTJADFFEKCKEDPSHWD |
| Motif 3 | 2.6e-506 | 50 | VFNVVILSPHGYFGQDBVLGLPDTGGQVVYILDQVRALETEMLLRINQQG |
| Motif 4 | 1.9e-496 | 50 | GQFRWIAAQTBRVRNGELYRYIADTKGAFVQPALYEAFGLTVVEAMTCGL |
| Motif 5 | 1.7e-465 | 50 | RVVHGIDVFDPKFNIVSPGADMSIYFPYTEEERRLTKFHPSIEELLYSEE |
| Motif 6 | 5.6e-464 | 50 | GKPDLIIGNYSDGNLVASLMAHKLGVTQCTIAHALEKTKYPDSDIYWKEL |
| Motif 7 | 5.1e-442 | 50 | EHLGYLADKKKPIJFSMARLDRVKNJTGLVEWYGKNKRLRELVNLVVVGG |
| Motif 8 | 1.7e-427 | 50 | LQRIYECYTWKIYSERLLTLGGVYGFWKHVSKLQRLEKRRYJEMFYNLKF |
| Motif 9 | 7.0e-422 | 50 | TTCNQRLERVEGTEHSHILRVPFRTEKGILRKWISRFDVWPYLETFTZDA |
| Motif 10 | 3.3e-390 | 50 | LKTTQEAIVLPPFVALAVRPRPGVWEYVRVNVYDLSVEELTPTEYLRFKE |
| Motif 11 | 3.1e-272 | 41 | WANDEFALELDFEPFBATVPRPTLSSSIGNGVEFJNRHLSS |
| Motif 12 | 4.7e-315 | 50 | LSELPKETPYEEFEARFKEMGFEKGWGBTAERVLEMMRLLSDILZAPDPS |
| Motif 13 | 2.8e-230 | 50 | LTRVDSIRERLNDALSAQRNELLALLSRYVAKGKGJLQRNNLINEFEKLI |
| Motif 14 | 6.2e-222 | 41 | KDSLEPLLDFLRLHNHQGENLMLNERINTINKLQHSLMKAE |
| Motif 15 | 7.3e-152 | 21 | SKDREEKAEIKKMHDLIEKYK |
