# Supplementary material for: Sucrose synthase gene family in Brassica juncea: genomic organization, evolutionary comparisons, and expression regulation
Source: PeerJ. 2021 Mar 9;9:e10878. doi: 10.7717/peerj.10878 (PMC7953879; doi:10.7717/peerj.10878)
Supplement: Supplemental Information 5 [file peerj-09-10878-s005.docx]

**Table S5:**

**Sequence and GO annotation information of SUS gene family members in *B. juncea*.**

| Name | Gene ID | Accession number | GO:biological process | GO:cellular component | GO:molecular function |
| --- | --- | --- | --- | --- | --- |
| BjuSUS01 | BjuA009339 | MW370524 | GO:0046686\|response to cadmium ion;  GO:0009409\|response to cold;  GO:0009413\|response to flooding;  GO:0009749\|response to glucose;  GO:0001666\|response to hypoxia;  GO:0010555\|response to mannitol;  GO:0006970\|response to osmotic stress;  GO:0072708\|response to sorbitol;  GO:0009744\|response to sucrose;  GO:0009414\|response to water deprivation;  GO:0005985\|sucrose metabolic process | GO:0005829\|cytosol;  GO:0009506\|plasmodesma | GO:0016157\|sucrose synthase activity |
| BjuSUS02 | BjuB015313 | MW370525 | GO:0046686\|response to cadmium ion;  GO:0009409\|response to cold;  GO:0009413\|response to flooding;  GO:0009749\|response to glucose;  GO:0001666\|response to hypoxia;  GO:0010555\|response to mannitol;  GO:0006970\|response to osmotic stress;  GO:0072708\|response to sorbitol;  GO:0009744\|response to sucrose;  GO:0009414\|response to water deprivation;  GO:0005985\|sucrose metabolic process | GO:0005829\|cytosol;  GO:0009506\|plasmodesma | GO:0016157\|sucrose synthase activity |
| BjuSUS03 | BjuA047153 | MW370526 | GO:0046686\|response to cadmium ion;  GO:0009409\|response to cold;  GO:0009413\|response to flooding;  GO:0009749\|response to glucose;  GO:0001666\|response to hypoxia;  GO:0010555\|response to mannitol;  GO:0006970\|response to osmotic stress;  GO:0072708\|response to sorbitol;  GO:0009744\|response to sucrose;  GO:0009414\|response to water deprivation;  GO:0005985\|sucrose metabolic process | GO:0005829\|cytosol;  GO:0009506\|plasmodesma | GO:0016157\|sucrose synthase activity |
| BjuSUS04 | BjuO008945 | MW370527 | GO:0046686\|response to cadmium ion;  GO:0009409\|response to cold;  GO:0009413\|response to flooding;  GO:0009749\|response to glucose;  GO:0001666\|response to hypoxia;  GO:0010555\|response to mannitol;  GO:0006970\|response to osmotic stress;  GO:0072708\|response to sorbitol;  GO:0009744\|response to sucrose;  GO:0009414\|response to water deprivation;  GO:0005985\|sucrose metabolic process | GO:0005829\|cytosol;  GO:0009506\|plasmodesma | GO:0016157\|sucrose synthase activity |
| BjuSUS05 | BjuA023848 | MW370528 | GO:0001666\|response to hypoxia;  GO:0010431\|seed maturation;  GO:0005982\|starch metabolic process;  GO:0005985\|sucrose metabolic process | GO:0005829\|cytosol;  GO:0016020\|membrane;  GO:0009505\|plant-type cell wall;  GO:0042170\|plastid membrane | GO:0016157\|sucrose synthase activity |
| BjuSUS06 | BjuB037515 | MW370529 | GO:0001666\|response to hypoxia;  GO:0010431\|seed maturation;  GO:0005982\|starch metabolic process;  GO:0005985\|sucrose metabolic process | GO:0005829\|cytosol;  GO:0016020\|membrane;  GO:0009505\|plant-type cell wall;  GO:0042170\|plastid membrane | GO:0016157\|sucrose synthase activity |
| BjuSUS07 | BjuA036504 | MW370530 | GO:0010555\|response to mannitol;  GO:0009414\|response to water deprivation;  GO:0010431\|seed maturation;  GO:0005982\|starch metabolic process;  GO:0005985\|sucrose metabolic process | N/A | GO:0016157\|sucrose synthase activity |
| BjuSUS08 | BjuO006586 | MW370531 | GO:0010555\|response to mannitol;  GO:0009414\|response to water deprivation;  GO:0010431\|seed maturation;  GO:0005982\|starch metabolic process;  GO:0005985\|sucrose metabolic process | N/A | GO:0016157\|sucrose synthase activity |
| BjuSUS09 | BjuA018844 | MW370532 | GO:0080165\|callose deposition in phloem sieve plate;  GO:0005985\|sucrose metabolic process | GO:0005618\|cell wall;  GO:0005576\|extracellular regio | GO:0016157\|sucrose synthase activity |
| BjuSUS10 | BjuB022852 | MW370533 | GO:0080165\|callose deposition in phloem sieve plate;  GO:0005985\|sucrose metabolic process | GO:0005618\|cell wall;  GO:0005576\|extracellular regio | GO:0016157\|sucrose synthase activity |
| BjuSUS11 | BjuA043452 | MW370534 | GO:0080165\|callose deposition in phloem sieve plate;  GO:0005985\|sucrose metabolic process | GO:0005618\|cell wall;  GO:0005576\|extracellular regio | GO:0016157\|sucrose synthase activity |
| BjuSUS12 | BjuB030220 | MW370535 | GO:0080165\|callose deposition in phloem sieve plate;  GO:0005985\|sucrose metabolic process | GO:0005618\|cell wall;  GO:0005576\|extracellular regio | GO:0016157\|sucrose synthase activity |
| BjuSUS13 | BjuB030962 | MW370536 | GO:0080165\|callose deposition in phloem sieve plate;  GO:0005985\|sucrose metabolic process | GO:0005618\|cell wall;  GO:0005576\|extracellular regio | GO:0016157\|sucrose synthase activity |
| BjuSUS14 | BjuB047347 | MW370537 | GO:0080165\|callose deposition in phloem sieve plate;  GO:0005985\|sucrose metabolic process | GO:0005618\|cell wall;  GO:0005576\|extracellular regio | GO:0016157\|sucrose synthase activity |
